# Design, Simulation, Fabrication, and Characterization of an Electrothermal Tip-Tilt-Piston Large Angle Micromirror for High Fill Factor Segmented Optical Arrays

**DOI:** 10.3390/mi12040419

**Published:** 2021-04-12

**Authors:** David Torres, LaVern Starman, Harris Hall, Juan Pastrana, Sarah Dooley

**Affiliations:** 1 Air Force Research Laboratory, Sensors Directorate, WP-AFB, Dayton, OH 45433, USA; lavern.starman.1@us.af.mil (L.S.); harris.hall.3@us.af.mil (H.H.); sarah.dooley@us.af.mil (S.D.); 2 Department of Electrical & Computer Engineering, Michigan State University, East Lansing, MI 48840, USA; pastran3@msu.edu

**Keywords:** MEMS, micromirror, COMSOL, large angle beamsteering, electrothermal, bimorph actuator, surface micromachining, finite element method, MEMS modeling

## Abstract

Micro-electromechanical system (MEMS) micromirrors have been in development for many years, but the ability to steer beams to angles larger than 20° remains a challenging endeavor. This paper details a MEMS micromirror device capable of achieving large motion for both tip/tilt angles and piston motion. The device consists of an electrothermal actuation assembly fabricated from a carefully patterned multilayer thin-film stack (SiO_2_/Al/SiO_2_) that is epoxy bonded to a 1 mm^2^ Au coated micromirror fabricated from an SOI wafer. The actuation assembly consists of four identical actuators, each comprised of a series of beams that use the inherent residual stresses and coefficient of thermal expansion (CTE) mismatches of the selected thin films to enable the large, upward, out-of-plane deflections necessary for large-angle beamsteering. Finite element simulations were performed (COMSOL v5.5) to capture initial elevations and tip/tilt motion displacements and achieved <10% variance in comparison to the experiment. The measured performance metrics of the micromirror include tip/tilt angles of ±23°, piston motion of 127 µm at sub-resonance, and dynamics characterization with observed resonant frequencies at ~145 Hz and ~226 Hz, for tip/tilt and piston motion, respectively. This unique single element design can readily be scaled into a full segmented micromirror array exhibiting an optical fill-factor >85%, making it suitable for optical phased array beam control applications.

## 1. Introduction

MEMS micromirrors have been widely developed for several decades in both academia and industry for a wide range of applications. During this significant period of time, many groups focused on single element designs while other researchers focused their efforts on closely packed arrays of micromirrors. Numerous micromirror devices have been successfully integrated into a vast array of commercial systems to include projectors [1], TVs [2], and optical scanners [3], just to name a few. However, in the past 10 years, several optical applications have arisen in which large-angle beamsteering can be both beneficial and required to meet new operational applications in areas such as medical instrumentation [4,5], LIDAR [6,7], projection display [8], smart windows [9], and free-space optical communications [10,11]. These devices can be classified into two groups by their actuation degrees of freedom: Tip (1D) or tip and tilt (2D), where most of the 2D devices are also capable of piston displacement. The digital micromirror devices (DMD) from Texas Instrument [1] are an example of 1D micromirror where the device can only move in one direction (tilt). In a two-state digital manner, 2D micromirrors (tip/tilt) are capable of scanning an area with a single element [12,13,14], typically in a continuous (or highly discretized near continuous) manner. However, few 2D devices presented in the literature [14] are scalable for arrays and maintain piston authority for phase alignment while demonstrating arbitrary 2D optical steering >20°. One reason for this is the design trade-off between the different performance parameters, which can include degrees of freedom in the structure, actuation range, array scalability (high fill factor), speed, among other parameters. Many of the examples presented before focus on designing devices with one or two optimized parameters. This work focuses on a design capable of achieving large 2D optical displacement with piston authority and high fill factor at the expense of achieving high speeds.

There are different actuation mechanisms that have been used to generate motion on micromirror devices, including piezoelectric [15,16,17], electromagnetic [18,19,20], electrostatic [1,8,21,22,23], and electrothermal [24,25,26]. Additionally, there has been work on high energy density (HED) materials [27] such as vanadium dioxide (VO_2_) [13,28] and shape memory alloy (SMA) [29]. These HED materials can be categorized as non-linear electrothermal devices as they exploit a phase change triggered by exceeding a threshold temperature, usually through the use of integrated heaters. All of these actuation mechanisms are capable of producing large steering angles under AC excitation either by driving them near their mechanical resonant frequencies (i.e., at resonance) or below (i.e., sub-resonance). 

Each of these actuation mechanisms offers benefits and disadvantages when integrated with MEMS structures. For example, piezoelectric materials are capable of producing fast actuation with large forces but are not capable of producing large displacement. This can be overcome by designing devices that operate at resonance. An example of this is presented by Gu-Stoppel et al. [30], where a 1D mirror achieved an optical range of over 100°. Another consideration of piezoelectric design is the requirement of non-conventional materials such as lead zirconate titanate (PZT), thus increasing the complexity and compatibility of the fabrication process. Similarly, electromagnetic actuators require permanent magnet materials to supply a constant magnetic field across the structure, which can be integrated within the fabrication of the structure or added outside of the device (external). Both electrostatic and electrothermal actuation can be enabled using conventional materials and standard microfabrication processes that are compatible with the standard Integrated Circuit (IC) and MEMS methods of manufacture. Electrostatic actuation is capable of faster actuation but generates lower forces than electrothermal, where the electrothermal speed is limited by the thermal dynamics of the system. The lower forces in electrostatic actuation require special attention in the design to achieve large displacement, as shown in [1,23]. Additionally, electrostatic actuation consumes low input power (due to the low consumption of current) for full actuation but typically requires high voltages (>100 V) to generate the forces capable of producing large displacement, as presented by Ozdogan et al. [21]. In electrothermal actuators, low drive voltages can induce large forces and displacements [31], but they consume much higher currents than electrostatic actuators, which results in higher input power requirements. Unlike VO_2_, piezoelectric, and SMA actuators, which all experience hysteresis [28,32], electrothermal actuators are easier to control as they exhibit linear behavior. For these reasons, electrothermal actuation was selected as the actuation mechanism for this device.

In an effort to achieve a design capable of large angular motion in both tip and tilt while maintaining piston authority for optical phase control, we have developed a surface micromachined actuation system (undercarriage) similar to work presented in 2009 by Jia et al. [24]. This undercarriage is then integrated with an optically flat micro-mirror plate and post, yielding a micromirror element design suitable for dense arrays with optical fill-factors (the ratio of reflective area to total device area) exceeding 85%. Work reported in 2011 by Jia et al. [33] demonstrated a mirror and the actuation system fabricated from the same wafer using bulk micromachining on a silicon on insulator (SOI) wafer, achieving an array with a fill factor of 88% and an actuation range of ±25° (tip/tilt). Our design has two main differences in comparison to this work: (1) It can be fabricated on virtually any substrate allowing for on-chip back end of the line (BEOL) MEMS integration with complementary metal-oxide-semiconductor (CMOS) or other foundry processing and (2) the fabrication of the mirror is decoupled from the undercarriage allowing for a largely unconstrained optical coating design and subsequently more optimal tailoring of the reflective surface for a given application independent of the actuation scheme. 

This work focuses on the characterization of single micromirror elements in the sub-resonance region with dynamic characterization to localize the resonant peak for each actuation motion: Tip/tilt and piston. In addition, the effect of the bonded mirror on top of the undercarriage is studied by comparing the output displacement of the device with and without the bonded mirror. Section 2 introduces the details on the design and fabrication of the device, in addition to the experimental setup used for characterization. Section 3 is dedicated to the presentation and discussion of the simulation and experimental results. Section 4 summarizes this work in the conclusion and provides a look into our future research. 

## 2. Design and Methods 

### 2.1. Design

**Figure 1:**
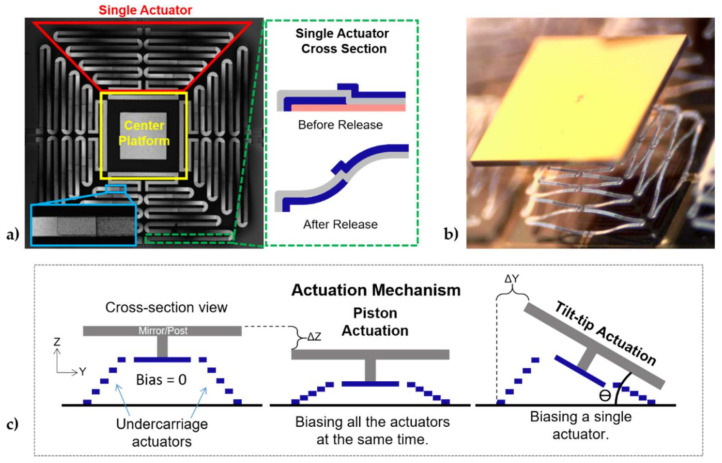
(**a**) Composite image from 3D confocal microscope of the actuation mechanism (undercarriage) of four actuators connected to a central platform with a footprint of 0.9 mm^2^. The left bottom inset shows the overlap (40 µm) between the layers on a single beam, and the right inset illustrates a cross-section of the actuators with oxide/metal/oxide layer configuration, (**b**) optical image of the released mirror-bonded device with a 1 mm^2^ mirror, and (**c**) representation of the actuation motions of the proposed device. The actuators start with an initial elevation, as the actuators are biased and the temperature is increased, the relaxation of the thermal film stresses from coefficient of thermal expansion (CTE) mismatch generates a downward motion, which results in a displacement towards the substrate. A piston-like motion is created when biasing all four actuators simultaneously with the same amplitude, while a tip/tilt motion is generated by applying a bias to a single actuator.

Two design parameters were selected as the focus of this micromirror design: (1) Large displacement for both tip/tilt and piston actuation, and (2) high fill factor for efficient use as an element in an optical phased array. The fill factor was defined as $\frac{\mathrm{Reflected}\;\mathrm{Area}}{\mathrm{Active}\;\mathrm{area}\;\mathrm{of}\;\mathrm{the}\;\mathrm{device}}\times 100$, where a fill factor of 100% means the reflected area is of the same size as the device and has no reflection losses. The displacement was dependent on both the actuator’s geometry/structure and the actuation mechanism. Hence, to achieve large displacement, the undercarriage design incorporated 4 electrothermal actuators integrated with a central platform to increase the overall output displacement (Figure 1a). The mirror and post structure were then bonded to the central platform of the undercarriage, increasing the fill factor of the element. The area of the mirror was 1 mm^2^ and the footprint of the undercarriage was selected to be 0.9 mm^2^. A fully assembled and released (mirror bonded) device is shown in Figure 1b. For the undercarriage design, the width of the beams was selected to be 20 µm to ensure mechanical stability, and a series of 8 beams were used between the anchor and the center platform. The length of the beams was selected to be as long as possible (within the footprint) to maximize the initial elevation, which in turn increased the overall actuation range. The thickness of the layers was first selected from modeling 2 pairs of beams connected in series to obtain the maximum initial elevation. The materials were selected to have a large CTE mismatch (Al and SiO_2_), which enabled a large actuation range over a set temperature range. Finally, the mirror thickness of 50 µm was selected to ensure a flat surface without compensation, and the post of 200 µm was selected to provide a clearance between the actuators and the mirror.

The thermal actuators are based on the S-shaped bimorph reported by Jia et al. [24], where the structure of the actuation system consisted of 3 stacked patterned thin film deposited layers of dielectric/metal/dielectric to create a series of bimorph beams, as shown in the inset of Figure 1a. The bimorph beams capitalized on the inherent residual stresses and the coefficient of thermal expansion (CTE) differences of the selected thin-film materials to create an upward, out-of-plane deflection, which was necessary to enable large-angle beamsteering. Biasing the actuators increased the temperature of the beams and generated a downwards force, resulting in a displacement towards the substrate. This was caused by the relaxation of the thermal stresses inherent to the elevated deposition temperatures and the CTE mismatches of the films created a downward motion. The design shown in Figure 1a has 4 pairs of cantilever-based designs connected in series to increase the overall displacement capability, where each pair was reduced in length as distance drew closer to the bonding platform. Each pair was connected in parallel with a mirrored pair of cantilever beams to form a single actuator. One can ascertain that each actuator can function highly independently from the other 3 actuators, thus enabling the tip/tilt motion needed to deflect the mirror by biasing a single actuator and piston motion when all 4 actuators were biased simultaneously with the same amplitude, as presented in Figure 1c. The bonded mirror had a 200 µm tall post that allowed the bonding between the mirror and the undercarriage without affecting the actuation mechanism, but it separated the rotational axis of the mirror. This created an inverse pendulum motion of the mirror during the tip/tilt actuation, resulting in a change of angle of the mirror and a lateral motion in the direction of the biased actuator, as illustrated in Figure 1c as a ΔY. 

One main advantage of the design was that the fabrication processing did not require high temperatures (>250 °C), allowing the possibility of direct BEOL integration of these devices with CMOS electronics [34]. This particular design was chosen to maximize the out-of-plane deflection while limiting the footprint to 1 mm^2^ to aid in micromirror array development with a 100% fill factor for a single element (reflecting element larger than the actuation scheme) and at least an 85.7% when used in an array. The mirrors in a 5 × 5 array were separated by a distance of 100 µm to avoid colliding with neighboring elements upon biasing a single actuator (as shown in Figure 1c), which resulted in a fill factor loss of 14.3%. The mirror was silicon coated with Au, which was highly reflective across a broad range of visible wavelengths, making it amenable for device testing. However, the mirror coating can be tailored to virtually anything, including optical metasurfaces, since the fabrication was independent of the undercarriage. While the mirror increased the fill factor of the device, it also increased the mass of the structure, affecting the dynamics of the undercarriage. The combination of the added mass of the mirror/post and the height of the post decreased the resonant frequencies of the structure and changed the dynamics of the system. One way of reducing the mass of the mirror without compromising the mirror curvature was by selectively removing material on the backside of the mirror, as reported by our group [35]. Reducing the height of the post decreases the moment of inertia and thus improved the dynamic performance. Additionally, this will also increase the fill factor of the array since it will need less separation between elements to avoid a collision. This work was a targeted demonstration effort, and the dimensions of the mirror structure presented in this work were selected to simplify the fabrication and bonding of the device as opposed to achieving optimal performance. More optimum geometries were possible, particularly with respect to post height. 

### 2.2. Fabrication

**Figure 2:**
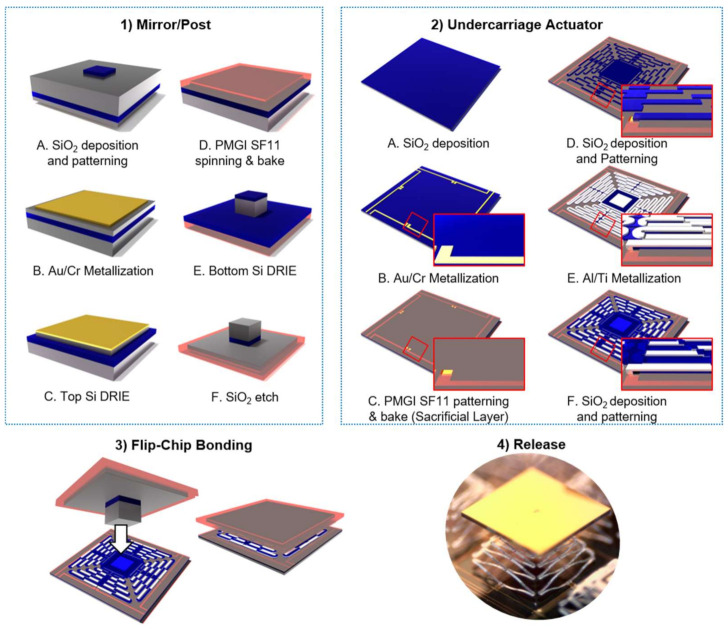
The fabrication process flow of the large angle micromirror device is composed of four steps: (1) Mirror/post-fabrication on a SOI wafer, (2) Undercarriage actuators fabrication using photoresist as the sacrificial layer, (3) Flip-chip bonding between the actuators and the mirror/post chip, (4) Release of the structure using solvent and a supercritical dryer (optical image).

The fabrication of the device is presented in Figure 2 and was divided into 4 steps: (1) Mirror/post-fabrication, (2) undercarriage fabrication, (3) flip-chip bonding, and (4) release. All lithography steps were patterned using a Karl Suss MA6 mask aligner. The mirror was fabricated on an SOI wafer with a handle thickness of 200 µm, a device layer of 50 µm, and 2 µm of buried silicon dioxide (SiO_2_). The fabrication of the mirror starts with the deposition and patterning of the SiO_2_ layer using a Plasma-Therm 790 plasma-enhanced chemical vapor deposition (PECVD) system on the backside of a doubled side polished SOI wafer. The oxide layer was used as a hard mask for the backside silicon (Si) deep reactive ion etch (DRIE). The DRIE was performed using an STS Pegasus 4 system. The metal layer of gold/chrome (200/20 nm) was patterned using standard lift-off processes on the top side of the wafer, creating the reflective layer of the mirror. All metals were evaporated using a Temescal BJD 2000. Chrome (Cr) was used as the adhesive layer between the Si and the gold (Au). A Si DRIE of 50 µm was performed on the top side of the wafer to define and divide each mirror element, using the buried oxide as the etch stop layer. A photoresist (PMGI SF11) was used on the top side of the wafer to protect the top metal layer and as a bonding agent between the elements of the array. Another Si DRIE (200 µm) on the backside of the wafer was performed to create the post of the mirror. Finally, the buried oxide was removed from the backside of the wafer with a reactive oxide ion etch (RIE). 

The undercarriage actuators were fabricated on a Si wafer. A 1 µm layer of SiO_2_ was used as an electrical isolation layer, followed by the metallization and patterning of the metal traces (Au/Cr layer) by lift-off. To start building the structural layers of the actuators, a sacrificial layer of photoresist (PMGI SF11) was spun and patterned to create the openings for the anchors. The following processes have a maximum temperature limit of 200 °C to avoid damaging the sacrificial layer. The subsequent layers were deposited and patterned to create the actuator structure: 1 µm SiO_2_ layer followed by a metal lift-off of Aluminum/Titanium (1/0.02 µm) and finished with a 1 µm SiO_2_ layer. The deposition conditions of the SiO_2_ are shown in Table A1. 

Once the mirror and the undercarriage were fabricated, they were bonded together with a flip-chip bonding tool from Finetech (Femto) using silver epoxy. The chips were manually aligned using the camera system within the bonding tool. The epoxy was prepared in a small container and placed by hand with the tip of a needle on the post of the mirror. The process took multiple experiments to achieve the desired quantity of epoxy on the chip, where a small amount of epoxy was not enough to hold the mirror and too much would spread across the actuators. A force between the mirror and the undercarriage was applied gradually until we reached a maximum of 0.5 N, then was held for 10 s. After the mirrors were bonded, the sample temperature was increased to 175 °C for 5 min. After the sample cooled down to room temperature, it was then placed inside an oven at the same temperature (175 °C) for another 5 min to cure the epoxy. The release of the structure was completed by submerging the samples in 1165 solvent at 95 °C for 20 min, followed by drying in a CO_2_ supercritical point dryer. An optical image of the released structure is presented in Figure 1b. Following the release of the fabricated devices, the structure exhibited an initial out-of-plane elevation due to the inherent residual stress and CTE of the materials. This initial elevation determined the maximum deflection range of the actuation system as the biasing of each actuator created a displacement towards the substrate, as illustrated in Figure 1c. 

The thickness of each layer of the undercarriage was measured and compared to the expected values as shown in Table 1. The layers were measured during the fabrication using a profilometer as a verification step for each process. The difference between the thicknesses of the layers was due to variance in the fabrication. One possible explanation for the difference in the top oxide layer can be attributed to over-etching of the layer as the thickness of the layer was verified on a witness wafer with an ellipsometer (Horiba Jobin Yvon UVISEL). This was caused by having a thinner layer of photoresist on the protected area due to the difference in heights created by the pattern on the wafer for the actuator from the previous layers. Additionally, the sacrificial layer of PMGI SF 11 was being removed by the over-etching of the bottom SiO_2_ layer, producing a false height measurement of the protective photoresist used for the top oxide. Although this over-etch occurred, through parametric simulations of this top oxide, it was determined that the thickness variation of the top oxide has minimal impact on the device’s initial displacement and actuation range until the oxide was less than 500 nm. The metal thickness of over 20% was caused by a higher deposition rate on the equipment. 

### 2.3. Experimental Setups

**Figure 3:**
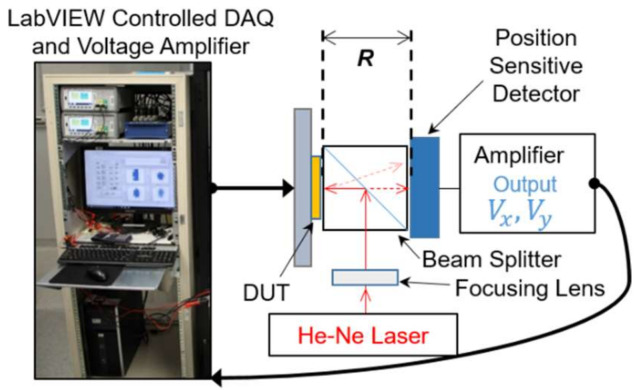
The optical setup used to characterize the tip/tilt angles of both devices, which allows one to measure the output range, step, and frequency response. The setup used a laser and a position sensitive detector (PSD) to track the tilting angle of the device, and the signals are controlled/read through LabVIEW DAQ, where *R* is the distance between the device under test (DUT) and the detector. The schematic is from a top view perspective.

An AC steady-state performance of both the undercarriage and the complete mirror-bonded device were characterized independently using the same procedures. First, the initial elevations of the structures were measured using a 3D confocal microscope (Olympus OLS4100, Olympus Corporation, Tokyo, Japan). This was followed by wire bonding the device to a package and measuring the electrical resistance of each actuator. Preliminary tests were done where the maximum current was determined to be 32 mA to avoid damaging the released structure with high temperatures. The dynamic response and the output range for tip/tilt actuation motion of the device were measured using the setup shown in Figure 3. In the setup, the device under test (DUT) was placed in front of a beam splitter, where a focused He-Ne laser was aligned to it and the reflection of the laser was then tracked using a position sensitive detector (PSD) from UDT Model 432 X-Y Position Indicator. The detector was connected to its amplifier, where the signal was amplified and converted into 2 voltages related to the position (V_x_, V_y_). These voltages were then converted into an optical angle of the device or laser position (frequency experiment) using the setup dimensions and basic trigonometry. LabVIEW was used to control all input signals and to collect all the experimental data using a National Instrument data acquisition (USB-6001). The setup was designed to capture the tip/tilt actuation motions of the device via reflected spot position by aligning a sensing laser normal to the mirror, minimizing the reading of the piston displacement on the sensor. This was achieved with the use of a beam splitter between the DUT and the sensor. The beam splitter increased the minimum distance between the DUT and the sensor to 41 mm (R) and limited the readable angles to 28.2° (optical angle). The angle measurements were limited by the geometry of the setup, as the laser will move out of the detector’s field of view with values larger than ± 28.2° (optical angle), given the distance *R* of 41 mm and a sensor diameter of 44 mm. For this reason, the optics between the DUT and the sensor were placed as close as possible to successfully capture the tip/tilt motion of the devices. 

The frequency response was performed only to the tip/tilt motion and measured using the setup from Figure 3. The experiments were performed by applying a sine wave as the input current to a single actuator where the amplitude was constant, and the frequency swept from 1 to 500 Hz, in steps of 1 Hz. This was then repeated for all the actuators of the devices. The frequency range was selected to be below the equipment’s limit response and within its cut-off frequency (<1 kHz). The input signal was verified with an oscilloscope to ensure the amplitude was constant as the frequency was increased. The output from the PSD was used to calculate the displacement magnitude (*DM*) as a function of frequency,

(1) $$DM\left(f\right)=\sqrt{{\left[X_{max}\left(f\right)-X_{min}\left(f\right)\right]}^{2}+{\left[Y_{max}\left(f\right)-Y_{min}\left(f\right)\right]}^{2}},$$

where *f* is the input frequency, *X* and *Y* values are the outputs from the PSD (position of the laser spot) converter to millimeter units for a particular frequency. The magnitude response as a function of frequency was calculated as,

(2) $$Magnitude\left(f\right)=DM\left(f\right)/I_{Mag}\left(f\right),$$

where *I_Mag_* is the input current magnitude. 

The piston actuation was measured using a commercial laser vibrometer (SIOS Nano Vibrometer Analyzer—NA Series). The vibrometer had an internal stabilized HeNe-laser and used interferometry to detect and record a range of vertical displacements. Considering that the vibrometer was integrated into a precision technical microscope, a 10X objective was used for a sensing laser spot diameter <10 μm. The laser spot was then aligned to the surface of the DUT (platform for the undercarriage and on the mirror for the mirror-bond device). To ensure the piston mode was effectively being actuated, the current of each actuator was controlled using LabVIEW, and the magnitude of the current was set to be the same between each actuator. At the moment of the measurement, the input and output collection were not synchronized. For this mode, the maximum current was less than for the previous one (e.g., tip/tilt), given that the thermal boundary conditions were different as the temperature is increasing on all the actuators at the same time. Additionally, the maximum displacement was reached at a lower input bias (e.g., current), as the mechanical resistance for displacement was reduced since all the actuators were working together. 

## 3. Results

### 3.1. FEM Model 

Finite element method (FEM) models for the undercarriage and the mirror-bonded devices were constructed in COMSOL 5.5 (COMSOL, Inc., Burlington, MA, USA). The material parameters for the model were taken from the COMSOL library (see Table 2) and the thickness for each layer was adjusted to the measured values. The model includes gravity to capture the effect of the added mass from the mirror structure. The initial deformation of the undercarriage was simulated by adjusting the volume reference temperature to 200 °C within the thermal expansion multiphysics module. This value corresponded to the deposition temperature of the oxide layers by PECVD. Additionally, to simulate heat transfer on the structure, multiple boundary conditions were added to the model as reported in [36]. First, the anchors were set to a constant temperature of 20 °C to capture the conduction through the anchors. Second, the heat dissipated to the environment was represented with two heat flux values: One for the upper surface to simulate the heat dissipated through the air to the environment and a second one for the bottom surface facing the substrate, where the heat is dissipated through the air to the substrate with values of 40 W/(m^2^·k) and 307.79 W/(m^2^·k), respectively. 

**Figure 4:**
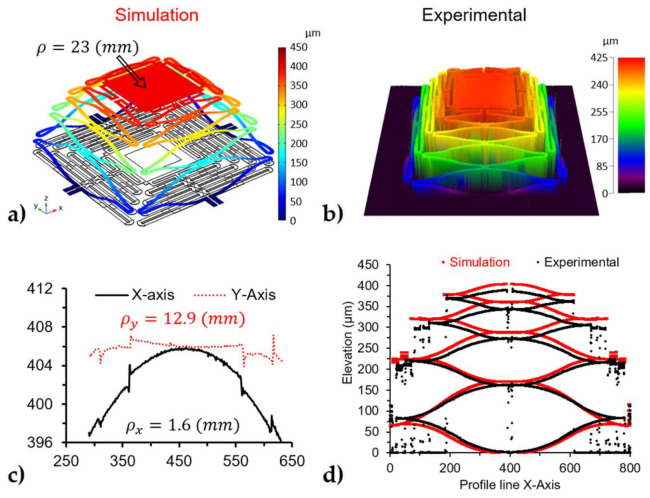
Initial elevation results of the undercarriage: (**a**) Simulation results from the COMSOL model, (**b**) experimental results from the released device (measured 3 months after the release), (**c**) profiles of the undercarriage platform measured at the center of the bonding platform to calculate the radius of curvature for both axis: X and Y, (**d**) comparison between the simulation and experimental results, where the profile of a single actuator is plotted together to illustrate the difference between the elevation and deformation of the results.

Figure 4a shows the steady-state simulation results without electrical biasing. The initial deformation was due to the residual stresses in the films as expected. The platform was elevated to 400 µm and had a symmetrical radius of curvature of about 23 mm. The simulation was then compared to the measurement of the undercarriage. The undercarriage was measured under the 3D microscope three months after release and the results are presented in Figure 4b. The samples were characterized three months after release to ensure steady-state from the temporal drift observed and were further explained in Section 3.2.1. Figure 4c measured the profile of the undercarriage platform with a resulting radius of curvature of 1.6 and 12.9 mm for the X and Y axis, respectively. The radius of curvatures on the platform was caused by the stresses of the film creating a cylindrical form on the square platform, as explained in Dunn et al. [37]. The stress in the material created a non-linear bifurcation of the plate that resulted in a cylindrical plate. The deformation profiles of each cantilever beam of the actuator were plotted together in Figure 4c for both the simulation and experimental results. The simulation results were in good agreement with the experimental results in both elevation and the shape of the deformation profile, with only a small difference (15.6 µm) in the total elevation of the actuator. The dissimilarity could be due to the material properties used in the COMSOL model. The properties for each material layer for the simulation were taken from the COMSOL 5.5 library, which come from bulk values of the material.

**Figure 5:**
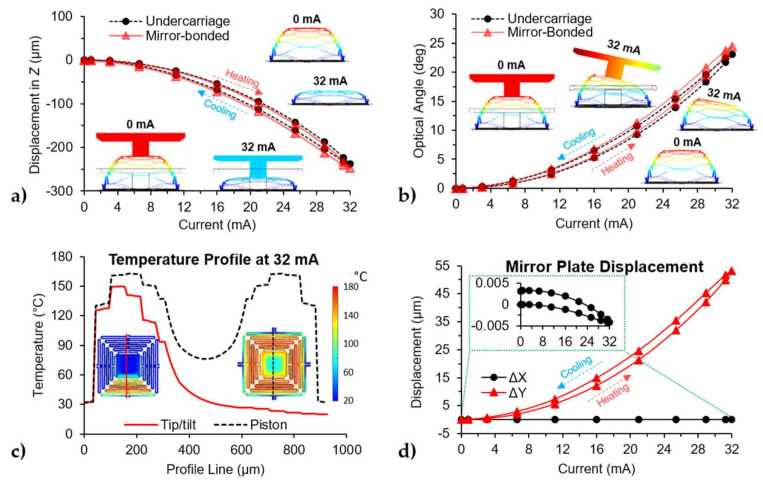
Time-based simulation results for the undercarriage and mirror-bonded device with a sine wave as the input: (**a**) Plots for piston displacement against input current with inset of the simulated devices at different times, where the input is applied to all four actuators at the same time, (**b**) plots for the optical angle against input current with insets of the simulated devices at different times, (**c**) plot for the temperature profile in the X-axis of the undercarriage for tip/tilt and piston actuation, insets show schematic of the undercarriage and the location of the profile line, (**d**) lateral displacement of the mirror plate as the device is actuated for tip/tilt motion.

Time-based simulations were performed for each actuation type (e.g., piston, tip/tilt) and for each device (e.g., undercarriage and mirror-bonded). In the simulations, the input bias current was a sine wave at 1 Hz sweeping from 0 to 32 mA for individual actuators. Figure 5a shows the displacement during piston actuation by applying the same input to all four actuators at the same time, while Figure 5b shows the tip motion or optical angle created by biasing a single actuator. Table 3 summarizes the output range of each actuation type, where the tilt values for the tip actuation captured the coupled cross-axis motion of the structure upon biasing a single actuator. The simulation demonstrates that the cross-axis motion for this design was negligible, i.e., the tip and tilt motions are decoupled from each other. Figure 5a,b shows that the mirror has a minimal effect on the undercarriage, as the mirror-bonded produced higher displacement with a range difference of 3.79% for the piston motion and 5.93% for the tip/tilt mode. The difference could be due to the mass of the mirror, which increased the moment of inertia for the tip/tilt and increased the force due to gravity in the piston motion mode. Additionally, both devices showed hysteresis on the output as they were actuated. One possible explanation for this behavior can be due to the difference in the heating/cooling rate of the structure. This asymmetry of the thermal dynamics of the system was caused by the geometry of the device as it takes longer to cool down than it takes to increase the temperature. This is shown in Figure 5a,b where the output of the respective plots was at a higher temperature when the input was decreasing compared to the same input value when the input was increasing. The corresponding temperature profiles at the same input value of the undercarriage for the tip/tilt and piston motion are presented in Figure 5c. As expected, the piston mode generated more heat with the same input values as all four actuators were biased at the same time. Although the actuators were largely thermally isolated (connected through SiO_2_), there existed some thermal conduction and thus temperature coupling between the actuators. Finally, Figure 5d shows the lateral displacement of the mirror plate as it is being actuated for tip/tilt motion with a maximum displacement of 53.15 µm. 

### 3.2. Characterization 

#### 3.2.1. Release Characterization

**Figure 6:**
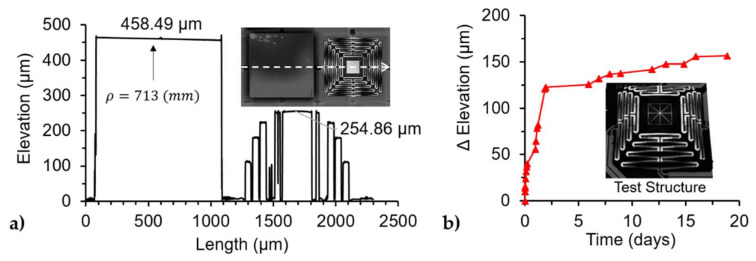
3D Confocal Microscope measurements: (**a**) Initial elevation of the mirror bonded and undercarriage device moments after release with an inset of the top view of the devices, (**b**) the change in test structure elevation over time with an inset of a composite image from the confocal microscope of the test structure. After the device was fabricated and released the elevation was tracked over a period of time without any external stimuli, and a temporal drift was observed.

Two devices were characterized and compared with each other: An actuation undercarriage and a complete mirror-bonded device. The initial elevation of the devices was measured after the structure was released, as shown in Figure 6a. The elevation of the devices was 458 µm and 254 µm for the mirror bonded device and the undercarriage, respectively. The platform in the undercarriage of the mirror-bonded device had an elevation of 193 µm. This was calculated by subtracting the thickness of the mirror, post, and epoxy. This thickness was obtained by measuring the mirror-bonded device before it was released with a total value of 265 µm. The 61 µm difference in the elevation of the platform between the two devices could be attributed to the mirror bonding process, where the combination of the applied force and the temperature could have affected the layers in the structure. In another observation, the elevation of the structures was changing over time without any biasing. To this end, a test structure from the same wafer was used to measure the changes in elevation as a function of time, and the results are shown in Figure 6b. The test structure had the same actuator design as the proposed device. It was observed that the device reached a steady state after a month. One possible explanation to this effect could be due to the quality of the oxide layer, where the SiO_2_ grown by PECVD at low temperature (200 °C) tends to react with the humidity in the environment creating a change in stress over time, as reported by [38,39,40]. This translates to a change of elevation over time in the undercarriage. Similar issues were reported by Wang et al. [41] and referred to as a temporal drift, where the structure of a piston only (without tip/tilt) device was designed with two sets of actuators connected in parallel with opposite motion direction (up/down) to minimize this effect. 

The undercarriage was characterized alone to observe the deformation on the actuators and used as a baseline to quantify the effects of the mirror on the undercarriage. The elevation of the DUTs was measured before each experiment, resulting in an elevation of 405 µm for the undercarriage and 592 µm for the mirror bonded device. The 3D image also captured the radius of curvature of each device for the undercarriage, as shown in Figure 4c, while the mirror shows a larger and symmetrical radius of curvature of about 71.3 cm for both axes. The curvature in the platform affected the laser reflection used in Figure 3, which tracked the displacement of the actuators.

#### 3.2.2. AC Steady-State

**Figure 7:**
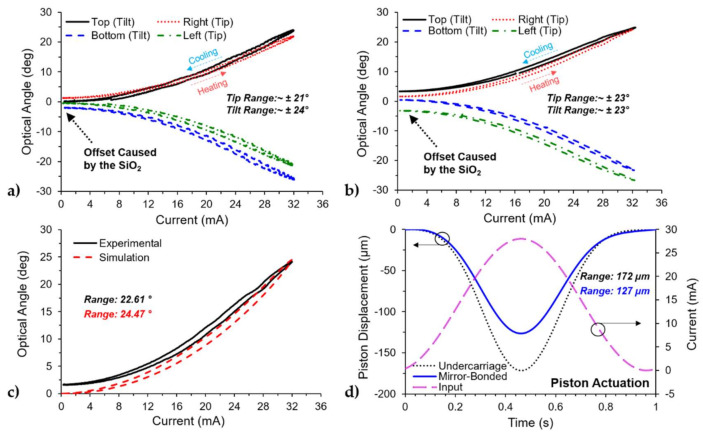
Measured AC steady-state performance (1 Hz) of both the undercarriage and the mirror-bonded device: (**a**) Tip/tilt motion for the undercarriage device, (**b**) tip/tilt motion for the mirror-bonded device, (**c**) comparison of the tip/tilt motion between experimental and simulation results of the mirror-bonded device, (**d**) piston motion for both devices.

The tip/tilt actuation was characterized under sub-resonant AC steady-state excitation conditions. A sine wave with an amplitude and offset value of 16 mA at 1 Hz was applied to each individual actuator, and measurements were done on both devices as seen in Figure 7a,b. The tilt angle was generated when either the top or bottom actuator was biased, where the top represented positive values and the bottom represented negative values. Similarly, the tip angles were generated when either the right or left actuators were biased, where the right represented positives values and the left represented negative values. The results for the undercarriage are shown in Figure 7a, where the noise in the plots was caused by the laser spot being distorted by the curvature of the undercarriage. Both devices show an offset between the measurements, as observed at the beginning of each plot. In [42], a similar structure with the same layers was reported to have non-linear behavior such as creep and fatigue, but we believe that the dominant effect shown in Figure 7a,b was caused by the low deposition temperature oxide and its reaction with the environment as reported by Haque’s group [39,40]. At room temperature, the water molecules in the environment interact with the oxide layer. When the actuator is biased and the temperature is increased, the water molecules are removed from the oxide layer. Once the temperature is lowered, the water molecules continue interacting with the oxide, changing the elevation over time, and moving the laser spot to a different location, thus creating an offset in the collected data. This offset was observed in the Y-Axis in the plots of Figure 7a,b. Furthermore, it was observed that the output range of the device was consistent over time when biasing a single actuator with a sinewave input current. 

Biasing individual actuators on the undercarriage achieved optical angles of about 21° and 24° for tip and tilt, respectively. The mirror bonded device achieved optical angles of about 23° for both tip and tilt. The results for the actuation range for each actuator are summarized in Table 4. The total optical angle range for the undercarriage was ~42° in the tip and ~48° in tilt. The difference in range between the tip and tilt was caused by the curvature on the reflective platform. The total optical angle range for the mirror-bonded device was ~46° for both tip and tilt. The mirror did not only increase the fill factor of the device, but it also increased the quality of the reflected laser resulting in a symmetrical beamsteering range. Additionally, the mirror itself did not affect the total range of actuation for the tip/tilt motion. Figure 7c compares the tilt motion (top actuator) of the mirror bonded device with the corresponding simulation. The simulation was able to capture the hysteresis behavior of the device and has a difference of 8.22% in the total range of the actuator. Additionally, while actuating in tip/tilt motion, there was a small displacement in the orthogonal direction. For example, biasing the top actuator that corresponded with tilt produced a tilt angle of 23.83° and tip angle of 1.05° or about 4% of the single actuation range. This small coupling between tip and tilt motion could be attributed to either misalignment in the optical setup or misalignment in the bonded mirror. Given the mass of the mirror/post structure, a misalignment in the mirror would create an asymmetry in the device that would generate an off-angle that would not be in the same direction as the biased actuator. 

The piston motion was triggered by biasing all four actuators simultaneously using a sine wave with an amplitude and offset of about 14 mA at 1 Hz, and the output was measured using the laser vibrometer. The results for the piston motion are shown in Figure 7d, where the actuation range for the devices was 172 µm for the undercarriage and 127 µm for the mirror-bonded. The difference in the range of 45 µm could be attributed to the initial elevation of the device, where the platform of the mirror-bonded device was 61 µm lower than the undercarriage alone, as a result of the bonding process. Finally, the piston displacement simulation shown in Figure 5a was able to capture the trend of the device but failed to capture the amplitude of the displacement when compared with the experimental results, as shown in Figure 7d. 

#### 3.2.3. Transient Response 

**Figure 8:**
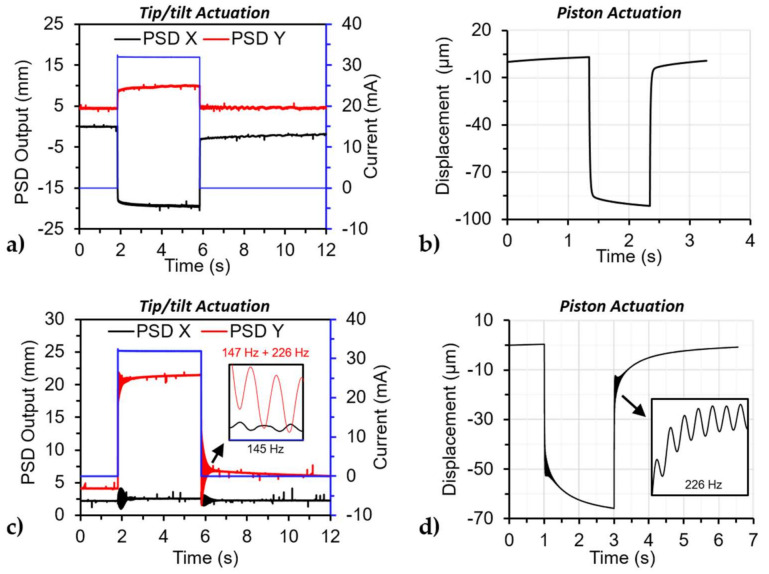
Step response for the undercarriage and the mirror-bonded device: (**a**) Tip/tilt motion for the undercarriage (top actuator), (**b**) piston motion for the undercarriage (right actuator), (**c**) tip/tilt motion for the mirror-bonded with an inset of a close-up look at the oscillation after the input reach 0 mA, and (**d**) piston motion for the mirror-bonded device with an inset of a zoom in of the oscillation.

The step response of the devices was done to capture the dynamics of the device for the tip/tilt and piston actuation motions. The step input was from 0 to 32 mA for the tip/tilt motion, while the maximum for the piston motion was 28 mA. The results for the step response are shown in Figure 8, where tip/tilt motion for the device had the same response for all the actuators. Figure 8a,c shows the step response for the top and right actuators of the undercarriage and the mirror-bonded device, respectively. Figure 8a shows motion perpendicular (PSD Y) to the biased actuator caused by the deformation on the platform of the undercarriage and quantified in Table 3. The output for the tip/tilt motion measurement is from the displacement of the laser spot in the sensor measured in millimeters, labeled as *PSD Output*. The output of the piston motion measurement was the displacement of the structure in the Z-axis in microns. The output values in the step response measurement on both devices and in both motions continued changing without reaching steady-state, as shown in Figure 8. This was attributed to the aforementioned behavior related to the PECVD oxide and observed in the tip/tilt and piston experiment. Due to this non-linear behavior, we were not able to calculate the time constant of the structures. However, it is clear that the added mass from the mirror changes system’s dynamics by reducing the damped resonant frequency of the system, as observed in Figure 8c,d. The oscillation frequency observed in the tip/tilt motion for the X-axis of the PSD was a combination of two frequencies ~147 Hz and ~226 Hz, while in the Y-axis of the PSD was ~145 Hz, as seen in Figure 8c. The frequency of 147 Hz corresponds to the tip motion while the 145 Hz corresponds to the tilt motion, additionally, the 226 Hz corresponds to the piston motion as evidenced by the frequency response results in Section 3.2.4. The resonant frequency difference between the tip and tilt motion can be attributed to the slight misalignment of the bonding process. Figure 8d shows the step response for the piston motion of the mirror-bonded device with an oscillation of 226 Hz, corresponding to the piston displacement. 

#### 3.2.4. Frequency Response 

**Figure 9:**
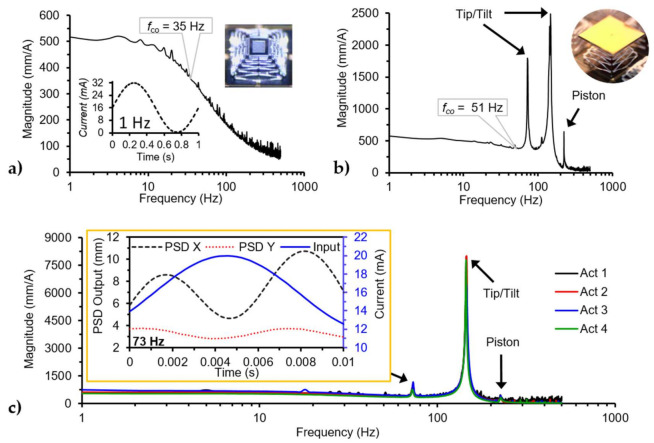
Frequency response for the undercarriage and mirror-bonded devices under single actuator bias. (**a**) Magnitude bode plot for the undercarriage with an inset of the input signal with a maximum value of 32 mA and another with an optical image of the undercarriage, (**b**) magnitude bode plot for the mirror-bonded device by applying an input current with a maximum value of 32 mA an inset of an optical mirror-bonded device, (**c**) magnitude bode plot for the mirror-bonded with an input current magnitude of 8 mA with an inset showing the input/output plot for 73 Hz, where the input and output frequencies were at 73 and ~145 Hz, respectively.

The frequency response for both devices is shown in Figure 9. The input applied to both devices was a sine wave with a magnitude of 32 mA (inset in Figure 9a), to a single actuator at a time (tip/tilt). The undercarriage did not present a resonant peak within the studied frequency range (Figure 9a), while the mirror-bonded device shows three resonant peaks at 73 Hz, 145 Hz, and 226 Hz (Figure 9b). Additionally, the cut-off frequency (*f_co_*) for the devices defined as the 0.707 of the DC magnitude was 35 Hz and 51 Hz for the undercarriage and the mirror-bonded device, respectively. The change in the cut-off frequency of the mirror-bonded device was the result of the new dynamic system created mainly by the mass of the mirror as a change in the moment of inertia of the system. 

A second experiment was performed on the mirror-bonded device with a lower input amplitude of 4 mA with an offset of 16 mA to better capture the resonant peaks, the results for each actuator are plotted in Figure 9c. The peak at 145 Hz comes from the tip/tilt mode and the 226 Hz peak comes from the piston mode. Finally, the peak at 73 Hz excites the mechanical mode for the 145 Hz, as shown in Figure 9c, where the input frequency was at 73 Hz, and the mechanical output was at 145 Hz. One explanation could be that the device was parametrically excited, as explained in [43]. The actuation mode agrees with the oscillation observed in the step response results. The resonant frequencies of the tip/tilt mode for each axis were very close to each other within the minimum resolution of the setup. The sub-resonance actuation range and dynamic results are summarized in Table 4. The output of the devices can achieve higher angles at resonance as the data collected at 145 Hz was limited by the optical setup, with a maximum readable optical angle range of 56.2°. 

## 4. Conclusions

The development and fabrication of an electrothermal MEMS beamsteering device capable of full tip/tilt and piston motion with integrated heaters while also exhibiting a high fill-factor (>85.7%) was achieved. A COMSOL model was able to capture the trend and magnitude for the initial elevation and tip/tilt motion with less than a 10% error. It was able the also capture the trend for the piston motion, but it was unsuccessful in capturing the magnitude of the motion. The large-angle micromirror device was created by fabricating the actuators and micromirror independently and then bonding them together to achieve a high-fill factor and enabling optical coating capabilities. In addition, the fabrication process for the undercarriage was done only by surface micromachining, which decreases the dependency of the substrate and increases the compatibility of the design with different technology such as CMOS and photonics processes. The characterization of the undercarriage and a mirror-bonded device was presented, where the mirror bonded device achieved an optical range of 46° at low frequency (sub-resonance). Comparison between devices demonstrated the effect of the mirror on the undercarriage, where the initial elevation was reduced by 61 µm, lowered the resonant frequencies of the system to the hundreds of hertz, and increased the cut-off frequency by 51%. 

Future research will consist of fabricating the undercarriage with different structural layers to address the non-linear effects from the oxide layers, continue studies on the resonant actuation, and continue developing the COMSOL model to capture the device actuation, including modal analysis, fully. Investigate the array design of the devices by characterizing the mechanical and thermal coupling effects between elements and the thermal effect of the traces on the actuators. Further, reducing the height and mass of the mirror without affecting the curvature on the mirror surface, will, in turn, shorten the dynamic response times and increase the device resonant frequencies making the device both more responsive and less susceptible to environmental vibration. These improvements would also reduce the lateral displacement of the mirror, allowing closer separation distances between the elements and higher fill factors for usage in arrays. Thus faster operation and more efficient optical power delivery seem feasible to achieve with this type of design.

## Figures and Tables

**Table 1:** Material layers thickness of the undercarriage structure.

Material Layer	Thickness Expected [µm]	Thickness Measured (µm)
Bottom Silicon Dioxide	1	1.02
Metal (Al/Ti)	1	1.19
Top Silicon Dioxide	1	0.64

**Table 2:** Material layers properties of the model structure used in the COMSOL 5.5 model.

Material Properties	Materials		
	SiO_2_	Al	Si
Thickness (µm)	1	1.2	0.6
Coefficient of Thermal Expansion (CTE) (10^−6^ 1/K)	0.5	23.1	2.6
Heat Capacity at Constant Pressure (J/(kg·K)	730	904	678
Density (kg/m^3^)	2200	2700	2320
Thermal Conductivity (W/m·K)	1.4	237	34
Young’s Modulus (GPa)	70	70	160
Poisson’s Ratio	0.17	0.35	0.22
Electrical Conductivity (GS/m)	N/A	35.5	N/A
Relative Permittivity	N/A	10	N/A

**Table 3:** Simulation results summary for the undercarriage and the mirror-bonded devices with a sine wave from 0 to 32 mA as the input signal.

	Biased Actuator	Undercarriage		Mirror-Bonded Device	
		Actuation Range		Actuation Range	
Tip Actuation ^1^	Single Actuator	Tip	Tilt	Tip	Tilt
		23.11°	0.00344°	24.48°	0.00655°
Piston Actuation	All	238.46 µm		247.57 µm	

^1^ Actuation range measured in optical angle (degrees).

**Table 4:** Experimental Results summary for the undercarriage and the mirror-bonded devices.

Motion	Biased Actuator	Undercarriage		Mirror-Bonded Device		
		Actuation Range ^1^		Actuation Range ^1^		Resonant Frequency (Hz)
		Tip	Tilt	Tip	Tilt	
Tip/Tilt	Top	3.08	24.34	1.47	21.75	145 ± 1
	Right	20.83	4.93	22.61	0.33	147 ± 1
	Bottom	5.1	24.19	1.05	23.83	145 ± 1
	Left	21.03	3.57	23.6	2.86	147 ± 1
Piston ^2^	All	172 µm		127 µm		226 ± 1
Cut-off Frequency ^3^		35 Hz		51 Hz		

^1^ Actuation range measured in optical angle (degrees) with an input magnitude of 32 mA. ^2^ Piston motion measured with an input magnitude of 28 mA. ^3^ Cut-off frequency was measured in tip/tilt motion.

## Appendix A

**Table A1:** Deposition condition for silicon dioxide grown by PECVD for the structural layer.

SiO_2_ Deposition Conditions	
RF Power (W)	25
Pressure (mT)	900
SiHe (sccm)	160
N_2_O (sccm)	900
Chamber Temperature (degC)	60
Electrode Temperature (degC)	200
DC Bias (V)	11
Time (min:sec)	15:20
Film Thickness (nm)	755.6
Film index	1.46
Deposition Rate (Å/min)	492.6
